# Genetic markers associated with insecticide resistance and resting behaviour in *Anopheles gambiae* mosquitoes in selected sites in Kenya

**DOI:** 10.1186/s12936-021-03997-4

**Published:** 2021-12-13

**Authors:** Sharon Mwagira-Maina, Steven Runo, Lucy Wachira, Stanley Kitur, Sarah Nyasende, Brigid Kemei, Eric Ochomo, Damaris Matoke-Muhia, Charles Mbogo, Luna Kamau

**Affiliations:** 1 Department of Biochemistry and Biotechnology, Kenyatta University, P.O Box 43844-00100, Nairobi, Kenya; 2 Centre for Biotechnology Research and Development, Kenya Medical Research Institute (KEMRI), P.O Box 54840-00200, Nairobi, Kenya; 3 Institute of Tropical Medicine and Infectious Diseases (ITROMID), P.O. Box 54840-00200, Nairobi, Kenya; 4 Centre for Global Health Research, KEMRI_CDC, P.O Box 1578-40100, Kisumu, Kenya; 5 KEMRI -Wellcome Trust Research Programme, Public Health Unit, P.O. Box 43640-00100, Nairobi, Kenya

**Keywords:** Insecticide resistance, *Kdr* mutation, *Ace-1*^*R*^ mutation, 2La inversion

## Abstract

**Background:**

Molecular diagnostic tools have been incorporated in insecticide resistance monitoring programmes to identify underlying genetic basis of resistance and develop early warning systems of vector control failure. Identifying genetic markers of insecticide resistance is crucial in enhancing the ability to mitigate potential effects of resistance. The knockdown resistance (*kdr*) mutation associated with resistance to DDT and pyrethroids, the acetylcholinesterase-1 (*ace*-1^*R*^) mutation associated with resistance to organophosphates and carbamates and 2La chromosomal inversion associated with indoor resting behaviour, were investigated in the present study.

**Methods:**

*Anopheles* mosquitoes sampled from different sites in Kenya and collected within the context of malaria vector surveillance were analysed. Mosquitoes were collected indoors using light traps, pyrethrum spray and hand catches between August 2016 and November 2017. Mosquitoes were identified using morphological keys and *Anopheles gambiae *sensu lato (*s.l*.) mosquitoes further identified into sibling species by the polymerase chain reaction method following DNA extraction by alcohol precipitation. *Anopheles gambiae* and *Anopheles arabiensis* were analysed for the presence of the *kdr* and *ace-1*^*R*^ mutations, while 2La inversion was only screened for in *An. gambiae* where it is polymorphic. Chi-square statistics were used to determine correlation between the 2La inversion karyotype and *kdr*-east mutation.

**Results:**

The *kdr*-east mutation occurred at frequencies ranging from 0.5 to 65.6% between sites. The *kdr-*west mutation was only found in Migori at a total frequency of 5.3% (n = 124). No *kdr* mutants were detected in Tana River. The *ace-1*^*R*^ mutation was absent in all populations. The 2La chromosomal inversion screened in *An. gambiae* occurred at frequencies of 87% (n = 30), 80% (n = 10) and 52% (n = 50) in Baringo, Tana River and Migori, respectively. A significant association between the 2La chromosomal inversion and the *kdr*-east mutation was found.

**Conclusion:**

The significant association between the 2La inversion karyotype and *kdr*-east mutation suggests that pyrethroid resistant *An. gambiae* continue to rest indoors regardless of the presence of treated bed nets and residual sprays, a persistence further substantiated by studies documenting continued mosquito abundance indoors. Behavioural resistance by which *Anopheles* vectors prefer not to rest indoors may, therefore, not be a factor of concern in this study’s malaria vector populations.

## Background

Malaria is one of the most prevalent vector-borne diseases in sub-Saharan Africa. According to the 2020 World Malaria Report, about 384,000 people in this region succumbed to the disease in the year 2019, most of them being children aged five years and below, and expectant mothers. Kenya accounted for 1% of deaths due to malaria globally [1]. Efforts towards malaria elimination in Kenya are focused on vector control and case management. The primary tools for malaria vector control in the country are Indoor Residual Spraying (IRS) and Long-Lasting Insecticidal Nets (LLINs). These interventions have been in use in the country for over twenty years, but their scaled-up utilization was only initiated in the 2000s. Insecticide-treated nets (ITNs) are distributed through ante-natal and child welfare clinics, comprehensive care clinics, designated rural shops, retail outlets and mass campaigns [2]. Kenya, being one among the countries most affected by the disease is listed under the US Government’s President’s Malaria Initiative (PMI) as a focus country for IRS- with the aim of limiting exposure to malaria vectors and reducing disease incidence and prevalence [3]. A number of studies show that the combined use of IRS and LLINs have led to a significant reduction in malaria morbidity and mortality in the country [4, 5]. However, these gains could be watered down by shifts in vector behaviour and the development of resistance to insecticides by the *Anopheles* mosquito vectors of malaria, coupled with resistance to anti-malarial drugs by *Plasmodium* parasites that cause the disease.

Genetic markers have been used for characterization of the molecular basis of insecticide resistance. Knockdown resistance (*kdr)* and acetylcholinesterase-1 (*ace-1*^*R*^) are single nucleotide polymorphic-type and restriction fragment length polymorphic-type biochemical markers, respectively, which detect changes in the amino acid sequence that brings about refractoriness to specific insecticides. *Kdr* for instance, confers resistance to pyrethroid and DDT insecticides, and is usually as a result of either a serine or a phenylalanine amino acid substituting a Leucine amino acid at locus 1014 of the voltage gated sodium channel gene (*Vgsc)* resulting in either an L1014S (*Vgsc*-1014S) or L1014F (*Vgsc*-1014F) mutation [6]. The *ace*-1^R^ mutation causes resistance to carbamates and organophosphates and results from a point mutation at 1ocus 119 where guanine is replaced with serine amino acid hence denoted as the G119S mutation [7]. The 2La inversion, denoted as 2La/2La, is one of the alternative arrangements of genes on the left arm of chromosome 2 and is a molecular marker associated with adaptation to different microclimates, desiccation resistance and mosquito behaviours [8]. Other arrangements include the standard arrangement and the heterokaryotype arrangement denoted as 2L+^a^/2L+^a^ and 2La/2L+^a^, respectively. While *Anopheles arabiensis* is fixed for this inversion karyotype, *Anopheles gambiae* remains highly polymorphic for the inversion. Since the different karyotypes are associated with different ecological conditions, the polymorphic *An. gambiae* has the advantage of ecological plasticity and this explains why it is widespread in Africa [9]. Genetic markers of insecticide resistance provide early warning of possible control failure in the future thereby enhancing the ability to cushion the possible negative effects of resistance on malaria vector control.

Resistance to various insecticides has been reported from multiple sites in Kenya [10]. It is likely that malaria incidence and mortality could substantially increase if insecticide resistance is left unchecked. Already, a rise in malaria prevalence has been reported at the Kenyan Coast even with the current malaria control strategies in place [11]. Indeed, insecticide resistance is one of the contributing factors towards the rise of malaria in areas where malaria prevalence had previously declined [12]. It is for this reason that the World Health Organization (WHO) through the Global Plan for Insecticide Resistance Management (GPIRM) calls upon the global malaria community to take urgent action to prevent an increase in insecticide resistance with the goal of ensuring that available vector control interventions remain effective. Insecticide resistance surveillance and management of any emerging resistance is a key strategy within this plan [13]. Understanding insecticide resistance mechanisms is vital for the rational management of insecticide resistance as it allows informed choice of replacement insecticides and their effective deployment [14]. In this study, we analysed *An. arabiensis* and *An. gambiae* mosquitoes sampled from selected sites in Kenya for the presence of two mutations associated with insecticide resistance. The first was the G119S (Glycine to Serine amino acid) mutation in the Acetylcholinesterase 1 (AChE-1) gene that is associated with resistance to organophosphate and carbamate insecticides [15]. The second was the L1014S or L1014F (Leucine to Serine or Leucine to Phenylalanine substitution) mutation at position 1014 of the voltage-gated sodium channel gene, that is associated with resistance to pyrethroids and organochlorines [16]. In addition, *An. gambiae* mosquitoes were analysed for the presence of the 2La chromosomal inversion that has been shown to be associated with an increased propensity for resting indoors at night where a saturation deficit exists [17]. This was so as to gain insights on behavioural adaptations that may impact the effectiveness of vector control interventions.

## Methods

### Study area

**Fig. 1:**
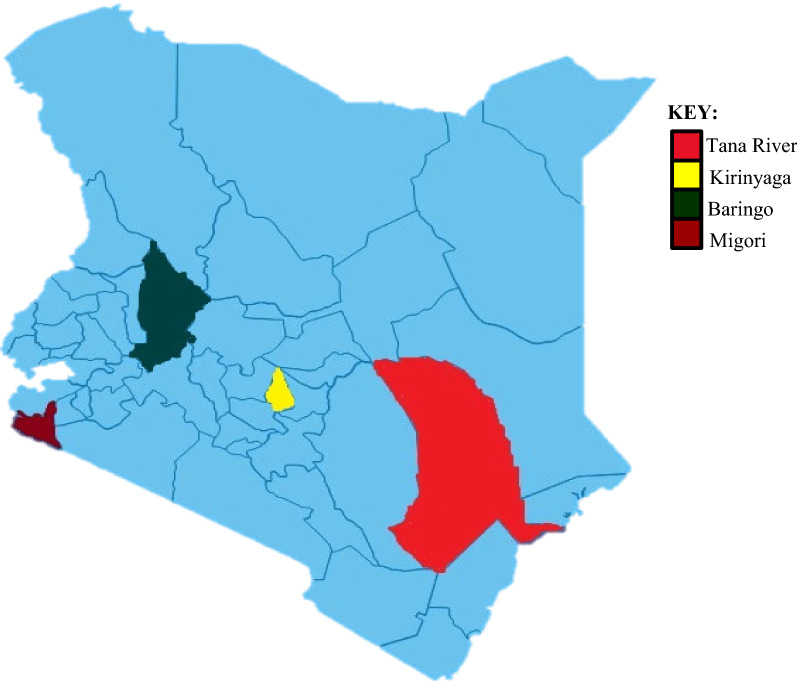
A Kenyan map showing Migori, Baringo, Kirinyaga and Tana River counties

The study was conducted in four sites in Kenya (Fig. 1) which are representative of different malaria epidemiological zones in the country. These were: Migori in the lake endemic region, Baringo in the highland epidemic region, Kirinyaga in the low risk malaria transmission zone and Tana River located in the seasonal transmission zone. The sites also vary in terms of vector species composition. Kirinyaga county has *An. arabiensis* as its predominant vector species with rare occurrence of *An. gambiae*, while Migori, Baringo and Tana River counties have these two target species coexisting.

### Mosquito specimens

Archived *Anopheles gambiae *sensu lato (*s.l*.) mosquitoes collected indoors using light traps and manual aspiration, between August 2016 and November 2017 within the context of a malaria vector survey conducted by Kenya Medical Research Institute (KEMRI) and the Kenya National Malaria Control Programme (NMCP) were used in this study. Light traps were set up inside selected houses at the foot side of the bed 1 m off the ground and were collected the following morning between 0600 and 0700 h. Indoor resting mosquitoes were collected using mouth aspirators between 0700 and 0900 h.

### Sample processing and identification

*Anopheles gambiae* (*s.l.*) mosquitoes were identified based on morphology [18] and were dissected into abdomen, legs, wings, head and thorax. Blood-fed abdomens, heads and thoraces were stored separately for other analysis. Genomic DNA was extracted from the legs, wings and unfed abdomens following the protocol of Collins et al. [19]. A portion of the extracted DNA was utilized in identification of *An. gambiae* and *An. arabiensis* sibling species, the two most predominant species in Kenya, using the polymerase chain reaction (PCR) assay [20].

### Genotyping of the *kdr and* of *ace-1*^*R*^ mutations

The *kdr* mutations in *An. gambiae* and *An. arabiensis* were analysed using real-time quantitative polymerase chain reaction (qPCR). A modified version of the protocol by Ochomo et al. [6] was followed. Real time-PCR reactions were run using a 96-well format on a Strata gene MxPro 3000 machine. Reaction curves for each set of reactions were visualized using Stratagene Mx3000P QPCR software and genotypes scored by eye. To detect the G119S mutation, a PCR-Restriction Fragment Length Polymorphism (RFLP) assay was conducted as described by Weill et al. [7]. The activity of the Alu I enzyme that was used in the restriction digest of amplified PCR-fragments was confirmed by restriction of Lambda DNA to expected fragments.

### Molecular karyotyping of the 2La chromosomal inversion

Presence of 2La chromosomal inversion was determined by the PCR assay of White and others as previously described [21].

### Data analysis

Data was analysed using STATA version 14.2 and Microsoft Excel version 10. Allele frequencies were generated for each molecular marker in the study sites using Microsoft Excel. The chi-square test was applied to determine whether allele frequencies varied significantly across different populations. Inversion genotypes and *kdr* genotypes were tested for their conformation to Hardy Weinberg equilibrium. Chi-square tests were used to determine association between the 2La inversion and the *kdr*-east mutation.

## Results

### Distribution of *An. gambiae* and *An. arabiensis* sibling species

A total of 731 *An. gambiae (s.l.)* mosquitoes were tested, with 666 specimens (representing 91.1%) successfully amplifying. Of the 666 specimens that were successfully amplified, 90.5% (n = 603) were identified as *An. arabiensis* and 9.5% (n = 63) as *An. gambiae*. Sixty-five specimens (representing 8.9%) of the total number of specimens tested failed to amplify. Both *An. arabiensis* and *An. gambiae* were found to occur in sympatry in the study sites at frequencies of 86.5% (n = 96) versus 13.5% (n = 15) in Baringo, 72% (n = 103) versus 28% (n = 40) in Migori and 95.2% (n = 157) versus 4.8% (n = 8) in Tana River, respectively. In Kirinyaga, only *An. arabiensis* was found. Table 1 is a summary of the *An. gambiae* (*s.l.)* sibling species distribution.

**Table 1:** Distribution of *An. gambiae* and *An. arabiensis* across study sites

Study site	N	*An. arabiensis* proportions (%)	*An. gambiae* proportions (%)
Baringo	111	86.5	13.5
Migori	143	72	28
Tana River	165	95	4.8
Kirinyaga	247	100	–
Mean	166.5	88.4	11.6

### Distribution of the *vgsc-1014S *and *vgsc-1014F kdr* alleles

A total of 308 samples were analysed for the *kdr* mutation. Table 2 summarizes the frequencies of *kdr* alleles in individual sibling species across the four study sites. The *vgsc-*1014S mutation occurred in both heterozygous and homozygous state with allele distributions conforming to Hardy–Weinberg expectations in all cases. The frequency of *vgsc-1014S* allele varied between species and across the different sites. *Anopheles gambiae* recorded a frequency of 65.6% (n = 32) and 9.4% (n = 32) in Migori and Baringo counties, respectively. Low frequencies (1.9% (n = 160) in Baringo and 0.5% (n = 196) in Kirinyaga) of the allele were observed in *An. arabiensis*. Tana River County had no record of the *vgsc-1014S* allele. The *vgsc-*1014F mutation was only recorded in Migori County in heterozygous state at a total frequency of 5.3% (n = 124). The distribution of the *vgsc-1014F* allele also varied between species with *An. gambiae *sensu stricto (*s.s*.) and *An. arabiensis* recording frequencies of 2.2% (n = 32) and 3.1% (n = 92), respectively.

**Table 2:** Frequencies of *kdr* alleles in *An. arabiensis* and *An. gambiae* in the four study sites

Study site	*An. arabiensis*			*An. gambiae*		
	N	L1014S (%)	L1014F (%)	N	L1014S (%)	L1014F (%)
Migori	92	–	3.1	32	65.6	2.2
Baringo	160	1.9	–	32	9.4	–
Kirinyaga	196	0.5	–	–	–	–
Tana River	94	–	–	10	–	–

L1014S: Leucine to Serine heterozygous *kdr* mutant

L1014F: Leucine to Phenylalanine heterozygous *kdr* mutant

### Distribution of the G119S mutation

The substrate DNA lambda used to test the activity of *Alu* I enzyme was successfully digested into multiple fragments, confirming the activity of the enzyme. PCR amplification of the expected 194 bp fragment was successful in 60 *An. gambiae* and 140 *An. arabiensis* specimens, respectively. However, none of the resulting PCR amplicons were digested by the *Alu* I enzyme, indicating absence of the G119S allele in all the study populations.

### Distribution of the 2La chromosomal inversion

Out of the 63 *An. gambiae* mosquitoes tested for the presence of the 2La chromosomal inversion, 45 successfully amplified. The 2La inversion allele occurred both in homozygous and heterozygous states and at allele frequencies of 87% (n = 30), 80% (n = 10) and 52% (n = 50) in Baringo, Tana River and Migori, respectively (Table 3). Compared to the heterokaryotype which was only found in Migori at 8% (n = 2), the homokaryotype inversion arrangement was more frequent and appeared at frequencies of 87% (n = 13) in Baringo, 80% (n = 4) in Tana River and 48% (n = 12) in Migori. As expected, the 50 (100%) *An. arabiensis* that were randomly selected for screening were found to be fixed for the 2La inversion homokaryotype.

**Table 3:** Distribution of allele and karyotype frequencies of the 2La inversion in *An. gambiae*

Site	No. specimens (n)	No. & % of standard karyotype 2L+^a^/2L+^a^	No. & % of inversion Heterokaryotype 2La/2L+^a^	No. & % of inversion homokaryotype 2La/2La	% 2La inversion allele
Baringo	15	2 (13%)	0	13 (87%)	87
Tana River	5	1 (20%)	0	4 (80%)	80
Migori	25	11 (44%)	2 (8%)	12 (48%)	52

### Association between the 2La chromosomal inversion polymorphism and the knockdown resistance (*kdr-*east) mutation in *An. gambiae*

Considering that the 2La chromosomal inversion has previously been found to be associated with the propensity for mosquitoes to rest indoors at night where a saturation deficit exists, the 2La inversion was used as a proxy for indoor resting behaviour. Chi-square tests were used to seek association between the 2La inversion karyotype and the *kdr-east* mutation, which was found to occur in significantly higher frequencies than the *kdr-west* mutation in our study populations. There was a significant association between the *kdr-east* mutation and the 2La inversion (Fisher’s exact test statistic value, F = 36.967, *P* = 0.000; Likelihood Ratio = 33.068, *P* = 0.000).

## Discussion

This study screened malaria vectors from selected sites in Kenya for the presence of the *kdr* and *ace-1*^*R*^ insecticide resistance markers, and 2La chromosomal inversion which has been associated with mosquito resting behaviour. It also sought to establish whether there is an association between markers of insecticide resistance and the 2La inversion and thus infer whether mosquito resting behaviour is associated with insecticide resistance. The study found an absence of the *ace-1*^R^ mutation in all the populations studied and a great variation in allele frequencies of *kdr* and inversion 2La across the sites. An association between the 2La inversion marker and the *kdr*-east mutation marker was observed.

A proper understanding of malaria vector species composition and other dynamics in a population informs the choice of vector control interventions with the greatest likelihood of success. This study reports sympatric occurrence of *An. gambiae* and *An. arabiensis* in Migori, Baringo and Tana River counties at varying frequencies, two species that have been described as the most efficient vectors of malaria [22]. Co-existence of several species of a complex in the same environment has previously been reported in many settings [23, 24]. In Kenya, *An. gambiae* is known to be common in western region [25–27]. This is reflected in Migori County in the western region where *An. gambiae* was found to occur in higher frequencies compared to the other study sites. The predominance of *An. arabiensis* in Kirinyaga and Baringo counties has been observed in previous studies which describe this sibling species as a dominant vector of the Central and Rift valley regions [28–30]. In Kirinyaga, only *An. arabiensis* was found. It is likely that this species is less affected by pyrethroid treated nets commonly used in the county as it preferentially feeds on cattle and is less endophilic [31, 32]. This gives it a competitive advantage over *An. gambiae,* which is endophagic, anthropophagic and endophilic in nature and is thus thought to be potentially killed by LLINs [33, 34]. Failure of a small number of the samples to amplify as either of the two sibling species could have been because of poor DNA quality or extremely low DNA concentrations. It is also possible that these samples belonged to other sibling species not targeted by the primers used in molecular identification. To ascertain the position, further testing with the relevant primers is necessary.

The 2La inversion is an important component of the natural malaria transmission system as it influences vector resting behaviour and susceptibility to *Plasmodium* [35]. In their studies for instance, Petrarca and Beier [36] observed that *An. gambiae* having the standard 2L+^a^/2L+^a^ karyotype were more susceptible to *Plasmodium* infection compared to those with the inverted 2La/2La homokaryotype. Since mosquitoes bearing the 2L+^a^ arrangement are behaviourally more exophilic, the finding that they are more *Plasmodium*-susceptible suggests the risk of their forming reservoirs of consistent outdoor malaria transmission. The 2La inversion has been associated with a higher propensity for indoor resting and a high frequency of this inversion in certain *An. gambiae* populations would thus mean that vector control applications targeting the indoor space would be more effective against such populations. The arid conditions of Baringo and Tana River seem to favour the occurrence of the 2La inversion, a situation which has been found to be the case in other studies [17, 22]. The high frequencies of the 2La inversion in these two regions suggest that most of the *An. gambiae* mosquitoes in these two regions remain inside human dwellings after blood feeding, consistent with known resting behaviour patterns of this species [37–39]. The implication of this observation is that vector control interventions that target indoor spaces, such as LLINs and IRS would be effective against *An. gambiae* in these regions. However, the decision on the choice of the intervention would need to be balanced against the fact that *An. gambiae* constituted only a small proportion of mosquitoes found in the study areas, with the outdoor resting *An. arabiensis* predominating.

Associations between inversion polymorphisms and insecticide resistance genes have previously been documented whereby loci within the inversion region on the left arm of chromosome 2 were found to be associated with insecticide resistance [39]. Although the *kdr* mutation is not located within the 2La inversion region, there is a significant association between the *kdr*-east mutation and the 2La inversion associated with indoor resting. This suggests that *An. gambiae* mosquitoes harbouring the *kdr*-east mutation still have a higher propensity to rest indoors suggesting that behavioural resistance may not be an important factor in this study population.

The *kdr vgsc-1014S* and *vgsc-1014F* alleles were found to occur both in *An. gambiae* and *An. arabiensis*. This observation is similar to other studies [6, 40] suggesting that both species have received exposure to vector control insecticides that cause selection pressure for knock-down resistance. The variation in frequency of these *kdr* mutations in *An. gambiae* and *An. arabiensis* could be due to environmental and behavioural attributes of these vector species [41]. Some mosquito breeding sites may for example, contain natural xenobiotics which larvae feed on. These compounds have been found to have an impact on the response of mosquitoes to pyrethroids through affecting mosquito metabolism which might also cross-select resistance mechanisms to pyrethroids thus modulating their insecticide tolerance [42, 43]. Outdoor feeding and resting are some behavioural attributes by which a species avoids contact with insecticides [44, 45]. Traits like these eventually influence the development of insecticide resistance and the frequency of *kdr* and other mutations associated with resistance. While some studies have reported the occurrence of *vgsc-1014S* in East Africa only [46, 47] and *vgsc-1014F* in West Africa only [47], others have reported the co-occurrence of both alleles in these regions [10, 48]. The *vgsc-1014F* allele was first documented in the country in 2012 [6] several years after its first report in West Africa. Other East African countries that have reported occurrence of the *vgsc-1014F* mutation include Uganda [49], Tanzania [50], Ethiopia [51] and Sudan [52]. Failure of the two mutations to comply with their geographical stratification as previously described indicate major shifts in *kdr* allele frequencies in malaria endemic countries and suggests gene flow between West Africa and East Africa [47, 53]. Although in the current study the *vgsc-1014F* mutation occurred at a very low frequency in Migori County, in concordance with previous studies [6, 41, 54], its presence in East Africa suggests that it is spreading. However, its occurrence remains widespread in West Africa [41].

In this study, we report the presence of the *vgsc-1014S* allele in Kirinyaga, Central Kenya which was not found in previous studies conducted in the region [55]. Although the allele was found at low frequencies in the current study, there is a possibility that frequencies could increase as a result of insecticide pressure from treated bed nets used as vector control tools in the area [56]. This would have a negative impact on pyrethroid vector control on this site. A possible explanation for the absence of *kdr* alleles in Tana River is that there is not a high enough buildup of insecticidal pressure to drive resistance genes. Some of the communities living in this region are nomadic pastoralists [57] and it is likely that their temporary house structures do not favor consistent deployment of insecticidal nets [58, 59]. Furthermore, the additional control methods relied upon such as burning cow dung and herbs to keep mosquitoes off houses have no insecticidal properties. The frequencies of *vgsc-1014S* in Baringo and Kirinyaga although low present a risk to the continued efficacy of pyrethroid-based vector control interventions. In Migori, the *vgsc-1014S* allele frequency was as high as 65.6% and is likely attributing to selection pressure by historic intensive pyrethroid spray programmes in the area. Having been classified as a stable malaria endemic county, Migori remained under intense pyrethroid-based IRS from the year 2010 to 2012 supported by the U.S. President Malaria Initiative [60]. Past plus present heavy use of pyrethroid-treated nets that are routinely distributed freely through antenatal and child welfare care clinics and mass campaigns are also likely contributing factors. Frequencies of the *kdr* mutations are likely to continue increasing and spreading in field mosquito populations as long as pyrethroid insecticide pressure from agricultural pesticides, bed nets and other interventions is present. This is likely to pose a great challenge to the effectiveness of control interventions that employ this insecticide class and others with the same mode of action. To counter this and preserve the efficacy of pyrethroid-based control interventions, there is need for the judicious use of insecticides, such as rotation in time and space of insecticides with different modes of action or their simultaneous use as mixtures [61].

The *ace-1*^*R*^ mutation was absent in the study populations. Although this mutation has been reported in several studies in West [41, 62] and Central Africa [63], there are no reports of its existence in Kenya. There is however need for monitoring the presence of this mutation especially in areas where organophosphates and carbamates may be in use for other purposes, such as in agricultural pests control [64]. The potential for this resistance mechanism to rapidly spread in *An. gambiae* was demonstrated through studies by Djogbénou et al. [62], in which a laboratory strain of *An. gambiae* homozygous for the *ace-1*^*R*^ mutation and code-named AcerKis was developed through introgression of the mutation from the insecticide-resistant *An. gambiae* (from Bobo-Dioulasso region of Burkina Faso) into the insecticide susceptible *An. gambiae* Kisumu strain in 2002. The absence of the *ace-1*^*R*^ allele in this study and the observed moderate phenotypic resistance to organophosphates and carbamates in the country [65, 66] may be associated with a metabolic resistance mechanism, such as overexpression of non-specific esterases (NSE) and elevation of detoxification enzymes.

## Conclusion

The significant association between the 2La inversion karyotype and *kdr*-east mutation suggests that pyrethroid resistant *An. gambiae* continue to rest indoors regardless of the presence of treated bed nets and residual sprays, a persistence further substantiated by studies documenting continued mosquito abundance indoors. Behavioural resistance by which *Anopheles* vectors prefer to not rest indoors may, therefore, not be a factor of concern in our study populations.

## Data Availability

The data analysed and used to make conclusions in this study are available from the corresponding author upon request.

## Abbreviations

Ace-1^*R*^: Resistant acetyl cholinesterase gene
AChE-1: Acetyl cholinesterase-1
Alu I: *Arthrobacter luteus* restriction enzyme
DNA: Deoxyribonucleic acid
GPIRM: Global Plan for Insecticide Resistance Management
G119S: Guanine to Serine amino acid substitution at locus 119
IR: Insecticide resistance
IRS: Indoor Residual Spraying
ITNs: Insecticide-treated nets
KEMRI: Kenya Medical Research Institute
Kdr: Knockdown resistance
LLINS: Long-Lasting Insecticidal Nets
NMCP: National Malaria Control Program
PCR: Polymerase chain reaction
PMI: President’s Malaria Initiative
RFLP: Restriction Fragment Length Polymorphism
s.l.: Sensu lato
s.s.: Sensu stricto
WHO: World Health Organization
Vgsc: Voltage gated sodium channel gene
2La/a: Inversion arrangement on left arm of chromosome 2
2L+^a^/+^a^: Standard arrangement on left arm of chromosome 2
2La/+a: Heterokaryotype arrangement on left arm of chromosome 2
